# Impacto Atual da Circulação Extracorpórea na Cirurgia de Revascularização Miocárdica no Estado de São Paulo

**DOI:** 10.36660/abc.20190145

**Published:** 2020-10-13

**Authors:** Gabrielle Barbosa Borgomoni, Omar Asdrúbal Vilca Mejia, Bianca Maria Maglia Orlandi, Maxim Goncharov, Luiz Augusto Ferreira Lisboa, Pedro Henrique Conte, Marco Antonio Praca Oliveira, Alfredo Inácio Fiorelli, Orlando Petrucci, Marcos Grandim Tiveron, Luís Alberto de Oliveira Dallan, Fabio Biscegli Jatene

**Affiliations:** 1 Universidade de São Paulo Universidade de São Paulo Faculdade de Medicina Hospital das Clínicas São PauloSP Brasil Universidade de São Paulo - Faculdade de Medicina Hospital das Clínicas - Instituto do Coração, São Paulo, SP - Brasil; 2 Hospital Samaritano PaulistaSão Paulo Hospital Samaritano Paulista São PauloSP Brasil Hospital Samaritano Paulista, São Paulo, SP - Brasil; 3 Beneficência Portuguesa de São PauloSão Paulo Beneficência Portuguesa de São Paulo São PauloSP Brasil Beneficência Portuguesa de São Paulo, São Paulo, SP - Brasil; 4 Universidade Estadual Campinas Universidade Estadual Campinas Faculdade de Ciências Médicas CampinasSP Brasil Universidade Estadual Campinas FCM Unicamp - Faculdade de Ciências Médicas, Campinas, SP - Brasil; 5 Hospital Santa Casa de Misericórdia Marília Hospital Santa Casa de Misericórdia Marília MaríliaSP Brasil Hospital Santa Casa de Misericórdia Marília, Marília, SP - Brasil

**Keywords:** Circulação Extracorpórea, Fatores de Risco, Revascularização Miocárdica, Epidemiologia, Mortalidade Hospitalar, Cuidados Pós-Operatórios, Morbidade

## Abstract

**Fundamento:**

Resultados prévios com o uso de circulação extracorpórea (CEC) geram dificuldades na escolha do melhor tratamento para cada paciente na cirurgia de revascularização miocárdica (CRM) no contexto atual.

**Objetivo:**

Avaliar o impacto da CEC no cenário atual da CRM no estado de São Paulo.

**Métodos:**

Foram analisados 2.905 pacientes submetidos à CRM de forma consecutiva em 11 centros do estado de São Paulo pertencentes ao Registro Paulista de Cirurgia Cardiovascular (REPLICCAR) I. Dados perioperatórios e de seguimento foram colocados via *on-line* por especialistas treinados e capacitados em cada hospital. Foram analisadas as associações das variáveis perioperatórias com o tipo de procedimento (com ou sem CEC) e com os desfechos. A mortalidade esperada foi calculada por meio do EuroSCORE II (ESII). Os valores de p menores de 5% foram considerados significativos.

**Resultados:**

Não houve diferença significativa em relação à idade dos pacientes entre os grupos (p=0,081). Dentre os pacientes, 72,9% eram de sexo masculino; 542 pacientes foram operados sem CEC (18,7%). Das características pré-operatórias, pacientes com infarto agudo do miocárdio (IAM) prévio (p=0,005) e disfunção ventricular (p=0,031) foram operados com CEC; no entanto, pacientes de emergência ou em classe funcional New York Heart Association (NYHA) IV foram operados sem CEC (p<0,001). O valor do ESII foi semelhante para ambos os grupos (p=0,427). Na CRM sem CEC, houve preferência pelo uso do enxerto radial (p<0,001) e com CEC pela artéria mamária direita (p<0,001). No pós-operatório, o uso de CEC esteve associado com reoperação por sangramento (p=0,012).

**Conclusão:**

Atualmente, no REPLICCAR, reoperação por sangramento foi o único desfecho associado ao uso da CEC na CRM. (Arq Bras Cardiol. 2020; 115(4):595-601)

## Introdução

A cirurgia de revascularização miocárdica (CRM) é um dos procedimentos mais estudados e, como consequência, foram alcançados excelentes resultados.^1^ O advento da circulação extracorpórea (CEC), sem dúvidas, permitiu estabelecer a CRM como um tratamento seguro, efetivo e reproduzível; no entanto, sempre existiu uma preocupação quanto à influência da CEC na morbimortalidade.^2^ As primeiras análises que compararam CRM com e sem CEC foram em pacientes de baixo risco e não mostraram diferença significativa.^3^ Com o tempo, o controle da CEC passou a apresentar melhoras, e começaram a ser encontrados benefícios a curto prazo unicamente para subgrupos de maior risco.^4^ Entretanto, estudos randomizados mais atuais que não encontraram diferenças a curto prazo revelaram problemas com a técnica sem CEC, em que apontam complicações relacionadas com a patência das anastomoses e com os maiores índices de revascularização incompleta.^5 - 7^ Assim, embora existam critérios bem-definidos para indicação da CRM, a escolha da CEC continua sendo baseada no perfil clínico do paciente e na experiência do cirurgião. A oportunidade atual para a técnica sem CEC pode estar no aumento de pacientes frágeis encaminhados para CRM,^8^ no conceito de levar o procedimento correto para o paciente correto. Por outro lado, resultados atuais da CRM com CEC mostram uma diminuição da ocorrência de acidente vascular encefálico (AVE),^9^ embora problemas com o aumento de sangramento e disfunção renal ainda persistam em pacientes de alto risco.

No Brasil, a proporção de pacientes submetidos à CRM sem CEC é variável, assim como os resultados de morbimortalidade.^10^ A falta de uma diretriz nacional que oriente o manuseio da CEC por meio de ações dirigidas por metas, protocolos de segurança e sistemas de monitoramento em tempo real pode estar influenciando nos resultados; portanto, existe uma lacuna na nossa realidade sobre o impacto da CEC na CRM.

O objetivo deste estudo foi avaliar o impacto atual da prática da CEC na morbimortalidade dos pacientes submetidos à CRM do REPLICCAR, o maior registro de cirurgias cardiovasculares do estado de São Paulo.

## Métodos

### Amostra

A amostra total foi de 5.222 pacientes, dos quais 2.905 foram submetidos à CRM nas 11 instituições participantes do estudo REPLICCAR.^11^ Os pacientes foram operados consecutivamente, de novembro de 2013 a dezembro de 2016, nos seguintes hospitais:

1. Instituto do Coração do Hospital das Clínicas da Faculdade de Medicina da Universidade de São Paulo (HC-FMUSP)
2. Beneficência Portuguesa de São Paulo
3. Hospital de Clínicas da Universidade Estadual de Campinas (Unicamp)
4. Irmandade da Santa Casa de Piracicaba
5. Irmandade da Santa Casa de São Paulo
6. Hospital Paulo Sacramento de Jundiaí
7. Hospital Pitangueiras do Grupo Sobam
8. Hospital das Clínicas de Ribeirão Preto
9. Hospital São Paulo da Universidade Federal de São Paulo (UNIFESP)
10. Hospital de Base de São José do Rio Preto
11. Irmandade da Santa Casa de Marília

## Critérios de Inclusão e Exclusão

### Critérios de Inclusão

Todos os pacientes com idade ≥ 18 anos, operados de CRM de forma eletiva, urgência ou emergência no período estabelecido.

### Critérios de Exclusão

Foram excluídos pacientes que realizaram, no mesmo procedimento, cirurgias associadas (incluindo valva, aorta torácica e outros) e procedimentos alternativos da insuficiência coronariana ( *laser* , injeção de células-tronco e outros).

## Coleta, Definição e Organização dos Dados

A análise foi realizada no banco de dados do REPLICCAR I. Trata-se de um registro prospectivo, multicêntrico e mandatório com coleta de dados entre novembro de 2013 e dezembro de 2016. A coleta de dados foi realizada por pessoas graduadas e treinadas para esta finalidade em cada centro participante. Os dados foram incorporados no *site* http://bdcardio.incor.usp.br, por meio de quatro interfaces disponíveis: pré-operatório, intraoperatório, pós-operatório e avaliação em 30 dias. O seguimento dos pacientes foi realizado por via telefônica. A completude e veracidade dos dados foram supervisionadas pelo comitê executivo do registro. As definições das variáveis foram adotadas a partir do EuroSCORE II, e o cálculo da mortalidade esperada no *site* http://www.EuroSCORE.org/calc.html.

Os desfechos analisados foram: morbidade (reoperação por sangramento, choque cardiogênico, AVE, infecção de sítio cirúrgico, mediastinite, pneumonia, infarto agudo do miocárdio [IAM] e insuficiência renal aguda [IRAg]) e mortalidade cirúrgica no período entre a cirurgia e a avaliação em 30 dias, ou, do contrário, até a alta hospitalar.

## Análise Estatística

A análise estatística foi realizada com o uso do *software* STATA versão 13.1 (StataCorp, Texas, USA). Para avaliar a distribuição de normalidade dos dados, foi utilizado o Teste de *Shapiro Wilk* . As variáveis IMC (<18,5kg/m2: baixo; 18,5 a 24,9kg/m2: normal; 25 a 29.9kg/m2: sobrepeso e; ≥30kg/m2: obesidade), classificação NYHA (I e II; III e IV), EuroSCORE II (<5% e ≥5%), hemoglobina glicosilada (≤7% e >7%), fração de ejeção (<30%; 30 a 50%; ≥50%), hematócrito (<30% e ≥30%) e creatinina (≤1,4mg/dL e >1,4mg/dL) foram categorizadas como frequências absoluta e relativa, com o intervalo de confiança binomial de 95%. Variáveis contínuas foram avaliadas quanto à diferença pelo teste de *Mann Whitney,* devido à distribuição dos dados. Contudo, para a comparação de categorias, utilizamos os testes qui-quadrado ou exato de *Fisher* . As variáveis contínuas assimétricas foram descritas através de mediana e intervalo interquartil. As variáveis de desfecho (complicações pós-operatórias) foram analisadas por meio da regressão logística univariada, e foram expressas a razão de chances ( *odds ratio* ) e o intervalo de confiança de 95%. Os valores de *p* menores de 5% foram considerados significativos.

## Ética e Termo de Consentimento

Este trabalho foi uma subanálise dentro do projeto intitulado “ *Estratificação de Risco Cirúrgico como Instrumento de Inovação em Programas em Cirurgia Cardíaca no Sistema Único de Saúde do Estado de São Paulo* ” com registro *on-line* n° 9696 da Comissão de Ética para Análises de Projetos de Pesquisa (CAPPesq) do HC-FMUSP.

## Resultados

Foram avaliados 2.905 pacientes submetidos à CRM no período de estudo. Destes, 542 (18,7%) não fizeram uso de CEC durante o procedimento. Não houve diferença significativa com relação à idade entre os grupos analisados ( *p* =0,081), a mediana dos pacientes com CEC foi de 63 anos (56 a 69), e sem CEC mediana, de 64 (56 a 71); 72,9% foram do gênero masculino.

Na Tabela 1 , estão descritas as características pré-operatórias dos grupos avaliados. Embora, os grupos demonstrem homogeneidade, observamos uma alta prevalência de infarto prévio do miocárdio em ambos os grupos (> de 40%), sendo significativamente maior para o grupo de pacientes escolhidos para a cirurgia com CEC (p=0,005). Não houve diferença significativa em relação ao valor da mediana do EuroSCORE II (p=0,482) para ambos os grupos.

**Tabela 1:** Características pré-operatórias da amostra de pacientes submetidos à CRM com e sem CEC. REPLICCAR, São Paulo, 2019

CEC						
Características	Sim (n = 2.363)		Não (n = 542)		IC 95%	Valor de p
	N	%	N	%		
**Idade, mediana (IIQ)**	63 (56-69)*		64 (56-71)*		62,4 a 62,6	0,081†
**Gênero**					0,25 a 0,29	0,125‡
Masculino	1.737	73,5	381	70,3		
Feminino	625	26,5	161	29,7		
**Índice de massa corporal**					27,3 a 27,6	0,809‡
< 18,5	14	0,6	4	0,7		
18,5 a 24,9	709	30	173	31,9		
25 a 29,9	1.057	44,7	234	43,2		
≥ 30	583	24,7	131	24,2		
**Infarto prévio do miocárdio**	1.142	48,3	226	41,7	0,45 a 0,49	**0,005‡**
***Stent* prévio**	389	16,5	98	18,1	0,15 a 0,18	0,363‡
**Cirurgia cardíaca prévia**	36	1,5	4	0,7	0,01 a 0,02	0,157‡
**Insulinodependente**	382	16,2	96	17,7	0,15 a 0,18	0,381‡
**Doença pulmonar obstrutiva crônica**	17	0,7	4	0,7	0,004 a 0,01	0,963‡
**Angina classe funcional IV**	442	18,7	91	16,8	0,17 a 0,20	0,299‡
**NYHA**					0,32 a 0,36	0,917‡
I e II	1.562	66,1	357	65,9		
III e IV	801	33,9	185	34,1		
EuroSCORE II					0,07 a 0,09	0,482‡
< 5%	2.156	91,5	501	92,4		
≥ 5%	200	8,5	41	7,6		

*Mediana e intervalo interquartil (IIQ); †Mann Whitney; ‡Teste qui-quadrado ou exato de Fisher.

Na Tabela 2 , podemos observar que pacientes com hemoglobina glicosilada > 7% foram operados com CEC (p=0,008). Quando a limitação esteve relacionada com o coração, como são os pacientes com FE<50%, eles foram escolhidos para cirurgia com CEC (p=0,031). O tipo de intervenção não esteve associado com valores de hematócrito e creatinina para os vários pontos de corte analisados neste estudo.

**Tabela 2:** Avaliação pré-operatória da amostra de pacientes submetidos à CRM com e sem CEC. REPLICCAR, São Paulo, 2019

CEC						
Exames pré	Sim (n = 2363)		Não (n = 542)			Valor de p
	n	%	N	%	IC 95%	
**Hemoglobina glicosilada**					6,6 a 6,9	**0,008‡**
≤ 7%	784	68,0	159	77,0		
> 7%	369	32,0	47	23,0		
**Fração de ejeção**					56,5 a 57,3	**0,031‡**
< 30%	36	1,5	4	0,7		
30 a 50%	474	20,1	87	16,1		
≥ 50%	1853	78,4	451	83,2		
**Hematócrito**					39,9 a 40,3	0,218‡
≥ 30%	2284	96,7	518	95,6		
< 30%	79	3,3	24	4,4		
**Creatinina**					1,1 a 1,2	0,651‡
≤ 1,4 mg/dL	2049	86,7	466	86,0		
> 1,4 mg/dL	314	13,3	76	14,0		

‡Teste qui-quadrado ou exato de Fisher.

No que diz respeito aos fatores intraoperatórios, na Tabela 3 , verificamos que, quando escolhida a técnica sem CEC, em relação àquela com CEC, houve maior utilização do enxerto de artéria radial (p<0,001) do que enxerto de artéria torácica interna direita (ATID) (p<0,001); no entanto, o uso de enxerto de artéria torácica interna esquerda (ATIE) não teve associação significativa (p=0,276) com alguma das técnicas.

**Tabela 3:** Fatores intraoperatórios da amostra de pacientes submetidos à CRM com e sem CEC. REPLICCAR, São Paulo, 2019

CEC						
Intraoperatório	Sim ( *n* = 2363)		Não ( *n* = 542)		IC 95%	Valor de p
	n	%	n	%		
**ATIE**	2.221	94	516	95,2	0,93 a 0,95	0,276‡
**ATID**	282	11,9	30	5,5	0,09 a 0,12	<0,001‡
**Radial**	134	5,7	114	21	0,08 a 0,1	<0,001‡

‡Teste qui-quadrado ou exato de Fisher.

Com relação aos eventos pós-operatórios, na Tabela 4 , não identificamos associação significativa para a ocorrência de AVE em até 30 dias após a cirurgia, sendo a proporção semelhante entre os casos com e sem CEC ( *p* =0,473). O uso da CEC não esteve relacionado com a mortalidade cirúrgica ( *p* =0,761). No entanto, o uso da técnica com CEC esteve associado com reoperação por sangramento ( *p* =0,001), levando a um aumento do risco de sangramento de 6,2 vezes (B = 1,8, IC95% 0,41-3,23).

**Tabela 4:** Regressão logística univariada de complicações pós-operatórias da amostra de pacientes submetidos a cirurgia de Revascularização do Miocárdio com e sem CEC. REPLICCAR, São Paulo, 2019

Desfechos clínicos e mortalidade	Uso de CEC				
	Sim (n = 2363)	Não (n = 542)	OR	IC95%	Valor de p
**Reoperação por sangramento**	53 (2,2)	2 (0,4)	6,2	1,5 a 25,5	0,012
**Choque cardiogênico**	77 (3,3)	20 (3,7)	0,88	0,53 a 1,45	0,614
**AVE**	19 (0,8)	5 (0,9)	0,87	0,32 a 2,3	0,784
**Infecção do sítio cirúrgico**	286 (12,1)	55 (10,2)	1,2	0,9 a 1,7	0,203
**Mediastinite**	16 (0,7)	6 (1,1)	0,61	0,24 a 1,6	0,303
**Pneumonia**	163 (6,9)	30 (5,5)	1,3	0,85 a 1,89	0,251
**IAM**	38 (1,6)	12 (2,2)	0,72	0,37 a 1,4	0,330
**IRAg**	118 (5,0)	32 (5,9)	0,84	0,56 a 1,25	0,338
**Óbito**	102 (4,3)	25 (4,6)	0,93	0,57 a 1,46	0,761

OR: Odds ratio; AVE: acidente vascular encefálico; IAM: infarto agudo do miocárdio; IRAg: insuficiência renal aguda.

## Discussão

Evidências mostram que a diminuição da resposta inflamatória na CRM sem CEC leva a uma redução de disfunção orgânica,^2^ assim como menores índices de vasoplegia e lesão renal.^12^ Neste cenário, análises retrospectivas em grandes populações confirmam uma redução significativa da morbimortalidade quando a CRM é realizada sem CEC.^13 , 14^ Assim, mesmo uma análise nos quatro maiores centros dos EUA mostrou benefício quando a CRM foi realizada sem CEC, principalmente nos pacientes considerados de alto risco.^15^ Dois estudos publicados no mesmo período, um nos EUA^16^ e outro no Brasil,^17^ revelaram também um maior risco de óbito nos pacientes operados com CEC em comparação aos operados sem CEC, principalmente no grupo de alto risco. Da mesma forma, uma análise de 30 anos da CRM sem CEC demonstrou uma redução significativa nos desfechos de mortalidade hospitalar, acidente cerebrovascular, complicações pós-operatórias graves, tempo de hospitalização e diminuição de custos.^18^

No entanto, estudos randomizados de grande impacto não mostraram diferença a favor da CRM sem CEC no que diz respeito à morbimortalidade.^19 - 21^ Em nossa análise, com uma amostra atual e multicêntrica, a única diferença encontrada a favor da CRM sem CEC foi o menor número de reoperações por sangramento. Isso também foi verificado no estudo de Lamy et al.,^22^ onde mesmo não existindo uma diferença significativa na morbimortalidade, houve uma diminuição na taxa de reoperação por sangramento. A taxa de reoperação por sangramento encontrada em nosso estudo é similar à encontrada em outro estudo,^23^ mas, em contrapartida, a mortalidade foi 4,5 vezes maior nos pacientes que tiveram essa complicação.

A prevalência de CRM sem CEC na nossa amostra é similar aos valores relatados em outras casuísticas,^24^ sugerindo a aderência às diretrizes na escolha da técnica e a inclusão de todos os pacientes no registro. Na análise, também foi evidenciado que, quando as limitações cirúrgicas estiveram relacionadas à manipulação do coração, como infarto prévio e/ou disfunção ventricular, a equipe tendeu a escolher a técnica com CEC. No entanto, bons resultados também foram encontrados quando operados sem CEC.^25^ Por outro lado, quando as limitações estiveram relacionadas com a gravidade do paciente, tipo NYHA IV ou estado de emergência, a escolha foi pela técnica sem CEC. Isso corrobora com estudos que mostram uma preferência pela CRM sem CEC nos pacientes instáveis.^26^ A maior utilização da artéria radial na CRM sem CEC pode ser explicada pelo menor tempo para obtenção do enxerto em relação à dupla mamária principalmente nos quadros agudos.

Cantero et al.,^27^ publicaram uma mortalidade hospitalar de 4,3% e 4,7%, respectivamente, no grupo sem CEC e com CEC (p=0,92), similar aos valores encontrados no nosso estudo (p=0,76). No entanto, diferentemente do nosso estudo, eles encontraram nos pacientes operados sem CEC um menor índice de complicações em relação ao infarto (p=0,02) e ao uso de balão intra-aórtico (p=0,01).

Neste estudo, não encontramos correlação significativa no que diz respeito ao gênero feminino com maiores índices de desfechos negativos. Como descrito por Sá et al.,^28^ isso talvez esteja relacionado com o tamanho amostral dos diferentes estudos.

Os escores de risco são instrumentos de predição que ajudam pacientes e profissionais da saúde na tomada de decisões sobre o provável risco de complicações ou óbito. Em um estudo realizado no InCor-HC-FMUSP, foi encontrado um ponto de corte para o EuroSCORE e o 2000BP que ajudaria na tomada de decisões para o uso ou não da CEC na CRM.^17^ Em nosso estudo, foi utilizado o EuroSCORE II, o mesmo que subestimou a nossa mortalidade observada, o que contraindicaria sua utilização para a tomada de decisões na amostra estudada. Isso confirma as orientações da última diretriz europeia, em que o uso atual do ESII para predição de mortalidade após CRM fica contraindicado.^29^

As limitações deste estudo foram: (1) a influência das variações no manuseio da CEC e dos protocolos sem CEC utilizados em cada centro participante não foi analisada. (2) Não foram analisados aspectos importantes, como o uso de antiagregantes plaquetários e o uso de antifibrinolíticos nos pacientes submetidos à CRM. No entanto, sabemos que o uso de protocolos seguindo as evidências atuais têm reduzido consideravelmente o aumento do risco de sangramento.^30 , 31^

Em resumo, ensaios clínicos randomizados não encontraram, em curto prazo, reduções na morbidade e mortalidade demonstradas nos estudos observacionais quando a CRM foi realizada sem CEC. No futuro, o uso de uma CEC mais monitorada e em tempo real, incluindo gasometria *on-line* e terapia dirigida por metas, pode ressaltar as vantagens do uso da CEC. Finalmente, é importante reiterar que o estado da arte atual é que grupos multidisciplinares definam e escolham a técnica correta para o paciente certo.

## Conclusão

Pacientes escolhidos para CRM com CEC, foram os mais estáveis clinicamente, porém, os com pior função ventricular em comparação aos operados sem CEC. A reoperação por sangramento foi o único desfecho associado à prática atual da CEC na CRM no estado de São Paulo; contudo, tal complicação não influenciou no aumento do número de óbitos.

Contribuição dos Autores

Concepção e desenho da pesquisa: Borgomoni GB, Mejia OAV, Lisboa LAF, Conte PH, Oliveira MAP, Petrucci Junior O, Tiveron M; Obtenção de dados: Borgomoni GB, Mejia OAV, Conte PH, Oliveira MAP, Petrucci Junior O, Tiveron M, Dallan LAO, Jatene FB; Análise e interpretação dos dados: Borgomoni GB, Mejia OAV, Goncharov M, Lisboa LAF, Conte PH, Oliveira MAP, Fiorelli AI, Petrucci Junior O, Tiveron M, Dallan LAO; Análise estatística: Borgomoni GB, Mejia OAV, Orlandi BMM, Goncharov M; Redação do manuscrito: Borgomoni GB; Revisão crítica do manuscrito quanto ao conteúdo intelectual importante: Mejia OAV, Lisboa LAF, Dallan LAO, Jatene FB.
