# Supplementary material for: Impacto Atual da Circulação Extracorpórea na Cirurgia de Revascularização Miocárdica no Estado de São Paulo
Source: Arq Bras Cardiol. 2020 Oct 13;115(4):595–601. [Article in Portuguese] doi: 10.36660/abc.20190145 (PMC8386981; doi:10.36660/abc.20190145)
Supplement: Translation

# Translation: Current Impact of Cardiopulmonary Bypass in Coronary Artery Bypass Grafting in São Paulo State

**Authors:** Gabrielle Barbosa Borgomoni, Omar Asdrúbal Vilca Mejia, Bianca Maria Maglia Orlandi, Maxim Goncharov, Luiz Augusto Ferreira Lisboa, Pedro Henrique Conte, Marco Antonio Praca Oliveira, Alfredo Inácio Fiorelli, Orlando Petrucci, Marcos Grandim Tiveron, Luís Alberto de Oliveira Dallan, Fabio Biscegli Jatene

**Affiliations:** 1 São PauloSP Brasil Universidade de São Paulo - Faculdade de Medicina Hospital das Clínicas - Instituto do Coração, São Paulo, SP - Brasil; 2 São PauloSP Brasil Hospital Samaritano Paulista, São Paulo, SP - Brasil; 3 São PauloSP Brasil Beneficência Portuguesa de São Paulo, São Paulo, SP - Brasil; 4 CampinasSP Brasil Universidade Estadual Campinas FCM Unicamp - Faculdade de Ciências Médicas, Campinas, SP - Brasil; 5 MaríliaSP Brasil Hospital Santa Casa de Misericórdia Marília, Marília, SP - Brasil

**Keywords:** Extracorporeal Circulation, Risk Factors, Myocardial Revascularization, \epidemiology, Hospital Mortality, Postoperative Care, Morbidity

## Abstract

**Background:**

Previous results on the use of cardiopulmonary bypass (CPB) have generated difficulties in choosing the best treatment for each patient undergoing myocardial revascularization surgery (CABG) in the current context.

**Objective:**

Evaluate the current impact of CPB in CABG in São Paulo State.

**Methods:**

A total of 2905 patients who underwent CABG were consecutively analyzed in 11 São Paulo State centers belonging to the São Paulo Registry of Cardiovascular Surgery (REPLICCAR) I. Perioperative and follow-up data were included online by trained specialists in each hospital. Associations of the perioperative variables with the type of procedure and with the outcomes were analyzed. The study outcomes were morbidity and operative mortality. The expected mortality was calculated using EuroSCORE II (ESII). The values of p <5% were considered significant.

**Results:**

There were no significant differences concerning the patients’ age between the groups (p=0.081). 72.9% of the patients were males. Of the patients, 542 underwent surgery without CPB (18.7%). Of the preoperative characteristics, patients with previous myocardial infarction (p=0.005) and ventricular dysfunction (p=0.031) underwent surgery with CPB. However, emergency or *New York Heart Association* (NYHA) class IV patients underwent surgery without CPB (p<0.001). The ESII value was similar in both groups (p=0.427). In CABG without CPB, the radial graft was preferred (p<0.001), and in CABG with CPB the right mammary artery was the preferred one (p<0.001). In the postoperative period, CPB use was associated with reoperation for bleeding (p=0.012).

**Conclusion:**

Currently in the REPLICCAR, reoperation for bleeding was the only outcome associated with the use of CPB in CABG. (Arq Bras Cardiol. 2020; 115(4):595-601)

## Introduction

The coronary artery bypass graft (CABG) surgery is one of the most studied procedures and, consequently, excellent results have been achieved.^1^The advent of cardiopulmonary bypass (CPB) has, undoubtedly, allowed establishing CABG as a safe, effective, and reproducible treatment, although there has always been a concern about the influence of CPB on morbimortality.^2^The first analysis that compared CABG with and without CPB was carried out in low-risk patients and did not show any significant differences.^3^Over time, CPB control was improved and short-term benefits began to be found only for subgroups at greater risks.^4^However, more recent randomized studies that did not find short-term differences disclosed problems with the technique without CPB, where complications occurred related to the patency of anastomoses and with the highest rates of incomplete revascularization.^5 - 7^Thus, although the criteria for CABG indication are well defined, the choice is based on the clinical profile and the surgeon’s experience. The current opportunity for the technique without CPB might be related to the increased number of fragile patients referred for CABG,^8^based on the concept of applying the correct procedure to the correct patient. Current results for CABG with CPB show a reduction in the incidence of stroke,^9^although problems with an increase in bleeding and renal dysfunction persists for high-risk patients.

In Brazil, the proportion of patients who undergo CABG without CPB is variable, as are the morbimortality results.^10^The lack of a national guideline that recommends the handling of CPB through goal directed perfusion, security protocols, and real-time monitoring could be influencing the results; thus, there is a gap in our understanding of the impact of CPB on CABG.

The goal of this study was to evaluate the current impact of CPB on the morbimortality of patients undergoing CABG recorded in the REPLICCAR, the largest cardiovascular surgery registry in São Paulo State.

## Methods

### Samples

The total number of samples included 5222 patients, of which 2905 underwent CABG at the 11 participating institutions of the REPLICCAR study.^11^The patients were consecutively submitted to surgery from November 2013 to December 2016 at the following hospitals:

1. Instituto do Coração do Hospital das Clínicas da FMUSP
2. Beneficência Portuguesa de São Paulo
3. Hospital de Clínicas da UNICAMP
4. Irmandade da Santa Casa de Piracicaba
5. Irmandade da Santa Casa de São Paulo
6. Hospital Paulo Sacramento de Jundiaí
7. Hospital Pitangueiras do Grupo SOBAM
8. Hospital das Clínicas de Ribeirão Preto
9. Hospital São Paulo da UNIFESP
10. Hospital de Base de São José do Rio Preto
11. Irmandade da Santa Casa de Marília

### Inclusion Criteria

All the patients were aged ≥18 years old and underwent CABG either electively, urgently, or because of an emergency during the established period.

### Exclusion Criteria

Patients who underwent associated surgeries (including valve, thoracic aorta, and other surgeries) and alternative procedures to treat coronary insufficiency (laser, stem cell injections, and others) were excluded.

## Data Collection, Definition, and Organization

The analysis was based on the REPLICCAR I database, which is a prospective, multicenter, mandatory registry that includes data collected between November 2013 and December 2016. The data collection was performed by people who had a degree and were trained for this exact purpose in each participating center. The data were incorporated into the website http://bdcardio.incor.usp.br/, through 4 available interfaces, including preoperative, intraoperative, postoperative, and evaluation after 30 days. Patient follow-up was performed through telephone interviews. The completeness and veracity of the data were overseen by the executive records committee. The definitions of variables were adopted from EUROSCORE II and the mortality calculation was performed using http://www.EuroSCORE.org/calc.html.

The analyzed outcomes were: morbidity (reoperation for bleeding, cardiogenic shock, stroke, surgical site infection, mediastinitis, pneumonia, acute myocardial infarction, and acute kidney injury) and surgical mortality in the period between the surgery and the 30-day evaluation, or otherwise, discharge from the hospital.

## Statistical Analysis

The statistical analysis was performed using STATA software version 13.1 (StataCorp, Texas, USA). The *Shapiro-Wilk* test was used to evaluate data distribution normality. The variables: BMI (<18.5kg /m^2^low, 18.5-24.9kg /m^2^normal, 25-29.9kg /m^2^overweight and ≥30kg / m^2^obesity), NYHA Classification (I and II and III and IV), EuroSCORE II (<5% and ≥5%), glycosylated hemoglobin (≤7% and> 7%), ejection fraction (<30%, 30-50%, ≥50%), Hematocrit (<30% e; ≥30%) and creatinine levels (≤1.4mg / dL and> 1.4mg / dL) were categorized as absolute and relative frequencies, with the binomial confidence interval of 95%. Continuous variables were assessed for the difference using the *Mann-Whitney* test, due to the data distribution. However, the Chi-square or Fisher’s Exact tests were used for the comparison of categories. Asymmetric continuous variables were described as median and interquartile range. The outcome variables (postoperative complications) were analyzed using univariate logistic regression and odds ratios and 95% confidence intervals were expressed. P values < 5% were considered significant.

## Ethics and Consent Form

This study is a subanalysis of the project entitled “Heart Surgery Programs Innovation Using Surgical Risk Stratification at the São Paulo State Public Healthcare System” registered online under number 9696 at the Ethics Committee for Research Projects Analysis Extended Diagnosis Clusters (CAPPesq) of Hospital das Clínicas da Faculdade de Medicina Universidade de São Paulo (HCFMUSP).

## Results

A total of 2905 patients who underwent coronary artery bypass graft surgery were analyzed during the study period. Of these, 542 (18.7%) did not undergo cardiopulmonary bypass during the procedure. There were no significant differences concerning the patients’ age between the groups (p=00.81), as the median age of patients submitted to CPB was 63 years old (56-69), whereas in the group without CPB it was 64 years old (56-71). 72.9% of the patients were males.

Table 1 shows the preoperative characteristics of the evaluated groups, where the sample homogeneity was presented. A high prevalence of prior myocardial infarction were observed in both groups (>40%), however, it was significantly higher in the patients selected for the procedure with the use of CPB (p=0.005). There was no significant difference in relation to the median value of the EuroSCORE II (p=0.482) for both groups.

**Table 1:** Preoperative characteristics of the patients who underwent coronary artery bypass graft with and without CPB. REPLICCAR, São Paulo, 2019

	CPB					
Characteristics	Yes (n= 2363)		No (n= 542)		95% CI	p Value
	N	%	N	%		
**Age, median, IQR**	63 (56-69) *		64(56-71) *		62.4 – 62.6	0.081 †
**Gender**					0.25 – 0.29	0.125 ‡
Male	1737	73.5	381	70.3		
Female	625	26.5	161	29.7		
**BMI**					27.3 – 27.6	0.809 ‡
<18.5	14	0.6	4	0.7		
18.5–24.9	709	30	173	31.9		
25–29.9	1057	44.7	234	43.2		
≥ 30	583	24.7	131	24.2		
**Prior myocardial infarction**	1142	48.3	226	41.7	0.45 – 0.49	**0.005 ‡**
**Prior stenting**	389	16.5	98	18.1	0.15 – 0.18	0.363 ‡
**Previous heart surgery**	36	1.5	4	0.7	0.01 – 0.02	0.157 ‡
**Insulin-dependent diabetes**	382	16.2	96	17.7	0.15 – 0.18	0.381 ‡
**COPD**	17	0.7	4	0.7	0.004 – 0.01	0.963 ‡
**Functional class IV angina**	442	18.7	91	16.8	0.17 – 0.20	0.299 ‡
**NYHA**					0.32 – 0.36	0.917 ‡
I & II	1562	66.1	357	65.9		
III & IV	801	33.9	185	34.1		
**EuroSCORE II**					0.07 – 0.09	0.482 ‡
< 5%	2156	91.5	501	92.4		
≥ 5%	200	8.5	41	7.6		

BMI: body mass index; COPD: chronic obstructive pulmonary disease. * Median and interquartile range (IQR), † Mann Whitney, ‡ Chi-Square or Fisher’s exact test.

Table 2 shows that patients with glycosylated hemoglobin >7% underwent surgery with CPB (p=0.008). When the limitation was related to the heart, patients with ejection fraction <50% were chosen to undergo surgery with CPB (p=0.031). The type of intervention was not associated with hematocrit and creatinine levels in the various cutoffs analyzed in this study.

**Table 2:** Preoperative evaluation of the patients who underwent coronary artery bypass graft with and without CPB. REPLICCAR, São Paulo, 2019

	CPB				95% CI	p Value
Pre-operative exams	Yes (n=2362)		Yes (n=2362)			
	n	%	N	%		
**Glycosylated hemoglobin**					6.6 – 6.9	0.008 ‡
≤ 7%	784	68.0	159	77.0		
> 7%	369	32.0	47	23.0		
**Ejection fraction**					56.5 – 57.3	0.031 ‡
< 30%	36	1.5	4	0.7		
30 – 50%	474	20.1	87	16.1		
≥ 50%	1853	78.4	451	83.2		
**Hematocrit**					39.9 – 40.3	0.218 ‡
≥ 30%	2284	96.7	518	95.6		
< 30%	79	3.3	24	4.4		
**Creatinine**					1.1 – 1.2	0.651 ‡
≤ 1.4 mg/dL	2049	86.7	466	86.0		
> 1.4 mg/dL	314	13.3	76	14.0		

‡ Chi-Square or Fisher’s exact test.

The intraoperative factors ( Table 3 ) verified that when the off-pump technique was chosen, in relation to the on-pump, there was a greater use of radial artery grafting (p<0.001) over the right internal thoracic artery (RITA) (p<0.001); however, the use of the left internal thoracic artery (LITA) grafts was not significantly associated (p=0.276) with any of the techniques.

**Table 3:** Intraoperative factors of the patients who underwent coronary artery bypass graft with and without CPB. REPLICCAR, São Paulo, 2019

	CPB					
Intraoperative	Yes (n=2362)		No (n=542)			
	N	%	n	%	95% CI	p value
**LITA**	2221	94	516	95.2	0.93 – 0.95	0.276 ‡
**RITA**	282	11.9	30	5.5	0.09 – 0.12	<0.001 ‡
**Radial**	134	5.7	114	21	0.08 – 0.1	<0.001 ‡

‡ Chi-Square or Fisher’s exact test.

The postoperative events ( Table 4 ) did not identify a significant association with the occurrence of stroke up to 30 days after surgery, with a similar proportion between patients with and without CPB (p=0.473). The use of CPB was not related to surgical mortality (p=0.761). However, it was associated with reoperation for bleeding (p=0.001), leading to a 6.2-fold increased risk of bleeding (B=1.8, 95% CI, 0.41-3.23).

**Table 4:** Univariate logistic regression of the postoperative complications of patients who underwent coronary artery bypass graft with and without CPB. REPLICCAR, São Paulo, 2019

Postoperative events and mortality	CPB				
	Yes (n=2362)	No (n=542)	OR	CI 95%	p Value
**Reoperation for Bleeding**	53 (2.2)	2 (0.4)	6.2	1.5 - 25.5	0.012
**Cardiogenic shock**	77 (3.3)	20 (3.7)	0.88	0.53 - 1.45	0.614
**Stroke**	19 (0.8)	5 (0.9)	0.87	0.32 - 2.3	0.784
**Surgical site infection**	286 (12.1)	55 (10.2)	1.2	0.9 - 1.7	0.203
**Mediastinitis**	16 (0.7)	6 (1.1)	0.61	0.24 - 1.6	0.303
**Pneumonia**	163 (6.9)	30 (5.5)	1.3	0.85 - 1.89	0.251
**Acute myocardial infarction**	38 (1.6)	12 (2.2)	0.72	0.37 - 1.4	0.330
**Acute kidney injury**	118 (5.0)	32 (5.9)	0.84	0.56 - 1.25	0.338
**Death**	102 (4.3)	25 (4.6)	0.93	0.57 - 1.46	0.761

OR: Odds ratio

## Discussion

Evidence has shown that the decrease in inflammatory response in off-pump CABG results in a decrease in organic dysfunctions,^2^as well as lower rates of vasoplegia and kidney injury.^12^This retrospective analysis in large populations confirms a significant decrease in morbimortality when CABG is performed without CPB.^13 , 14^Furthermore, an analysis of the 4 largest centers in the United States of America (USA) has shown benefits when CABG is performed without CPB, mainly in high-risk patients.^15^Two studies published regarding the same time period, one in the USA^16^and the other in Brazil,^17^also revealed an increased risk of death in patients undergoing CABG with CPB compared with those without CPB, especially in the high-risk group. Similarly, an analysis of 30 years of CABG without CPB showed a significant decrease in hospital mortality outcomes, such as stroke, severe postoperative complications, hospitalization time, and cost reduction.^18^

Nonetheless, randomized high impact trials did not show any difference in favor of CABG without CPB regarding the morbimortality.^19 - 21^In our analysis, with a current sample and multicentric study, the only difference found in favor of CABG without CPB was the lower number of reoperations for bleeding. This was also verified in the analysis of Lamy et al.,^22^that when no significant difference occurred in morbidity and mortality, there was a decrease in the need for reoperation for bleeding. The rate of reoperation for bleeding found in this present study is similar to that found in another study,^23^but conversely, the mortality rate was 4.5 times higher in patients who had this complication.

The prevalence of CABG without CPB in our sample is similar to the values reported in other studies,^24^suggesting adherence to the guidelines regarding the choice of technique and the inclusion of all the patients in the registry. The analysis also evidenced that, when the surgical limitation was related to the heart manipulation, such as prior myocardial infarction and/or ventricular dysfunction, the group leaned towards choosing the technique with CPB. However, good results were also found when CABG was performed without CPB.^25^Conversely, when limitations were related to patient severity, NYHA IV or an emergency situation, the choice was off-pump surgery. This confirms the studies that showed a preference for CABG without CPB in unstable patients.^26^The higher utilization of radial arterial grafts in CABG without CPB can be explained by the shorter time required for graft preparation in relation to the double thoracic artery graft, mainly in acute cases.

Cantero el al.^27^reported a hospital mortality rate of 4.3% and 4.7% in a group without CPB and with CPB, respectively (p=0.92), similar values to those found in this study (p=0.76). However, the difference from this study is that patients submitted to surgery without CPB had fewer complications related to myocardial infarction (p=0.02) and the use of the intra-aortic balloon pump (p=0.01).

In this study, we did not find any significant correlation with the female gender and a higher index of negative outcomes, as described by Sá el al.,^28^which may be related to the sample size of the different studies.

The risk scores are prediction instruments that help patients and health professionals in their decision making about probable risks of complications or death. In a study conducted at InCor-HCFMUSP, a cutoff was found for the EuroSCORE and the 2000BP that would help in decision making to not use CPB with CABG.^17^This study uses EuroSCORE II, the same that underestimated our observed mortality, which would contradict its utilization in decision making regarding the studied sample. This confirms the recommendations of the last European guideline, where the total use of EII for the prediction of mortality after CABG is contraindicated.^29^

The limitations of this study are as follows: (1) The influence of variations in the handling of CPB and non-CPB protocols used in each participating center were not analyzed; (2) There were no important analyses, such as the use of antiplatelet agents and the use of antifibrinolytics in patients who underwent CABG. However, it is known that the use of protocols following current evidence has considerably reduced the increased risk of bleeding.^30 , 31^

In summary, randomized clinical trials did not find a short-term reduction in morbidity and mortality demonstrated in observational studies when CABG was performed without CPB. In the future, the use of a more monitored and real-time CPB, including online gasometry and goal-directed therapy, may highlight the advantages of using CPB. Finally, it is important to reiterate that the current state-of-the-art condition is that multidisciplinary groups define and choose the correct technique for the right patient.

## Conclusion

Patients chosen for CABG with CPB were the most clinically stable, but with worse ventricular function than those without CPB. Reoperation for bleeding was the only outcome associated with the current CPB practice in São Paulo State; however, this complication did not influence the increase in the number of deaths.
